# Distribution of antibiotic resistance genes and their pathogen hosts in duck farm environments in south-east coastal China

**DOI:** 10.1007/s00253-023-12842-4

**Published:** 2024-01-15

**Authors:** Kaidi Liu, Minge Wang, Yin Zhang, Chang Fang, Rongmin Zhang, Liangxing Fang, Jian Sun, Yahong Liu, Xiaoping Liao

**Affiliations:** 1 National Risk Assessment Laboratory for Antimicrobial Resistance of Animal Original Bacteria, South China Agricultural University, Guangzhou, People’s Republic of China; 2 School of Agricultural Science and Engineering, Liaocheng University, No.1 Hunan Road, Liaocheng, 252000 Shandong China; 3 Laboratory of Veterinary Pharmacology, College of Veterinary Medicine, South China Agricultural University, Guangzhou, 510642 People’s Republic of China; 4 Guangdong Provincial Key Laboratory of Veterinary Pharmaceutics Development and Safety Evaluation, South China Agricultural University, Guangzhou, 510642 China; 5 Guangdong Laboratory for Lingnan Modern Agriculture, Guangzhou, 510642 People’s Republic of China; 6 Jiangsu Co-Innovation Center for the Prevention and Control of Important Animal Infectious Diseases and Zoonoses, Yangzhou University, Yangzhou, 225009 People’s Republic of China

**Keywords:** ARGs, Duck farm, Environment, Bacterial community, Human bacterial pathogens

## Abstract

**Abstract:**

Livestock farms are major reservoirs of antibiotic resistance genes (ARGs) that are discharged into the environment. However, the abundance, diversity, and transmission of ARGs in duck farms and its impact on surrounding environments remain to be further explored. Therefore, the characteristics of ARGs and their bacterial hosts from duck farms and surrounding environment were investigated by using metagenomic sequencing. Eighteen ARG types which consist of 823 subtypes were identified and the majority conferred resistance to multidrug, tetracyclines, aminoglycosides, chloramphenicols, MLS, and sulfonamides. The *floR* gene was the most abundant subtype, followed by *sul*1, *tet*M, *sul*2, and *tet*L. ARG abundance in fecal sample was significantly higher than soil and water sample. Our results also lead to a hypothesis that Shandong province have been the most contaminated by ARGs from duck farm compared with other four provinces. PcoA results showed that the composition of ARG subtypes in water and soil samples was similar, but there were significant differences between water and feces samples. However, the composition of ARG subtypes were similar between samples from five provinces. Bacterial hosts of ARG subtypes were taxonomically assigned to eight phyla that were dominated by the *Proteobacteria*, *Firmicutes*, *Bacteroidetes*, and *Actinobacteria*. In addition, some human bacterial pathogens could be enriched in duck feces, including *Enterococcus faecium*, *Acinetobacter baumannii*, and *Staphylococcus aureus*, and even serve as the carrier of ARGs. The combined results indicate that a comprehensive overview of the diversity and abundance of ARGs, and strong association between ARGs and bacterial community shift proposed, and benefit effective measures to improve safety of antibiotics use in livestock and poultry farming.

**Key points:**

• *ARG distribution was widespread in the duck farms and surroundings environment*

• *ARG abundance on the duck farms was significantly higher than in soil and water*

• *Human bacterial pathogens may serve as the vectors for ARGs*

**Supplementary information:**

The online version contains supplementary material available at 10.1007/s00253-023-12842-4.

## Introduction

Antibiotics are critical for the prevention and treatment of bacterial infections and the spread of antibiotic resistance undermines their effectiveness (Dai et al. 2022). Antimicrobial resistance is a growing and major global health concern due to the high rates of morbidity and mortality that it can cause an estimated 10 million deaths by 2050 if unchecked (O'Neill 2014; Rodríguez et al. 2021). The excessive and indiscriminate use of antibiotics in both human and veterinary medicine has significantly accelerated the widespread development of antibiotic resistance. As a result, antibiotic resistance genes (ARGs) are now ubiquitous in the human-animal-environment interface (Li et al. 2021). In recent years, a deeper appreciation of the “One Health” concept has emerged, recognizing the links between the health of humans, animals, and the environment. This has led to the recognition that livestock and poultry farms and their surrounding environments are crucial links in the dissemination of antibiotic resistant organisms (Hooban et al. 2021). An increasing number of studies have demonstrated that swine feedlots and abattoirs, wild boars, fish, broiler chickens, and dairy farms are recognized the reservoirs of ARGs (Bai et al. 2021; Dias Diana 2021; He et al. 2019; Jo et al. 2021; Munk et al. 2018). However, the spread and distribution characteristics of ARGs in duck farms have been largely ignored. Duck production has the potential to play a major role in the agricultural economy, and Asian countries alone contribute 84.2% of total duck meat produced in the world. China is the largest global producer and consumer of cultivated ducks (Wang et al. 2021b). Thus, it is necessary to assess the ARG profile and distribution of duck farms in China.

Considerable evidence suggested that ARGs and antibiotics are released from livestock and poultry farms into their surroundings that include water, soil and air (Zhang et al. 2020c). For example, the airborne ARGs can disperse from the animal houses to a distance of 10 km along the wind direction in chicken and dairy farms (Bai et al. 2021). ARGs are also released from stored swine manure biogas digestate to the atmosphere via aerosol dispersion (Zhang et al. 2020c). These bioaerosols play keys roles in transmission of antibiotic resistance, and levels of airborne ARGs from pig farms are elevated during the winter months (Song et al. 2021). In addition, swine farming can enhance the levels of veterinary antibiotics and ARGs in groundwater (Gao et al. 2020). Furthermore, a lack of sewage treatment techniques in intensive breeding has led to ARG contamination of neighboring fishponds and aquaculture water and associated ARGs are discharged directly into rivers, lakes and seas without treatment (Fu et al. 2022; He et al. 2019). After that, ARGs can also persist in aquatic environments and this promotes further dissemination of ARGs between aquatic environment and aquatic animals (Jo et al. 2021; Mahaney and Franklin 2022).

Composting is an economical and environmentally friendly approach to treat cattle, swine, and chicken manure (Wang et al. 2022; Xu et al. 2022). The elevated temperatures produced during composting can decrease ARG abundance for some bacterial taxa, and some ARGs have proven recalcitrant to this process (Mao et al. 2021). Agricultural products can also be contaminated with ARGs through manure or to a lesser extent by compost and consequently are a potential health risk to both animals and humans (He et al. 2019). ARG migration to deeper soil layers by long-term land application of animal manures has increased the complexity of the problem (Tang et al. 2015). Therefore, ARG monitoring on duck farms and their surrounding environment is the first step into assessing whether this poses a health threat.

Bacterial community shifts are key factors driving changes of ARG profiles in different host habitats (Guo et al. 2021). Generally, swine manure is a diverse bacterial source and the genera *Romboutsia*, *Clostridisensu_stricto_1*, and *Terrisporobacter* are the primary bacterial ARG hosts found during swine manure composting (Wang et al. 2021a). However, the *Streptococcaceae* are also sources of multidrug, MLS, and aminoglycoside ARGs in pig feces (Zhang et al. 2021), and *Prevotellaceae* and *Ochrobactrum* are enriched with tetracycline ARGs in airborne samples (Song et al. 2021). Metagenomic assembly–based host-tracking analysis identified *Escherichia*, *Bacteroides*, and *Clostridium* as the predominant bacterial hosts of ARGs in gut-associated environments and pristine environments (Zeng et al. 2019). Our previous work indicated a major presence of carbapenemase-producing Enterobacteriaceae on duck farms and their surrounding environments (Wang et al. 2021c). Therefore, this prompted us to explore the roles of bacterial community shifts in the resistome alterations that originate on duck farms and surrounding environment.

In this study, duck feces and surrounding environmental samples were collected from duck farms in south-east coastal China including Shandong, Jiangsu, Zhejiang, Fujian, and Guangdong province. The main objectives of the study are to compare the diversity and abundance of ARGs in duck feces and their surrounding environment, correlate ARGs with the bacterial community and further predict the potential ARGs host, and explore ARG-associated human pathogenic bacteria. These findings will contribute to the full understanding of ARGs, ARG-hosts, and ARG-associated human pathogenic bacteria in duck farms and their surrounding environment.

## Materials and methods

### Sample collection

**Fig. 1:**
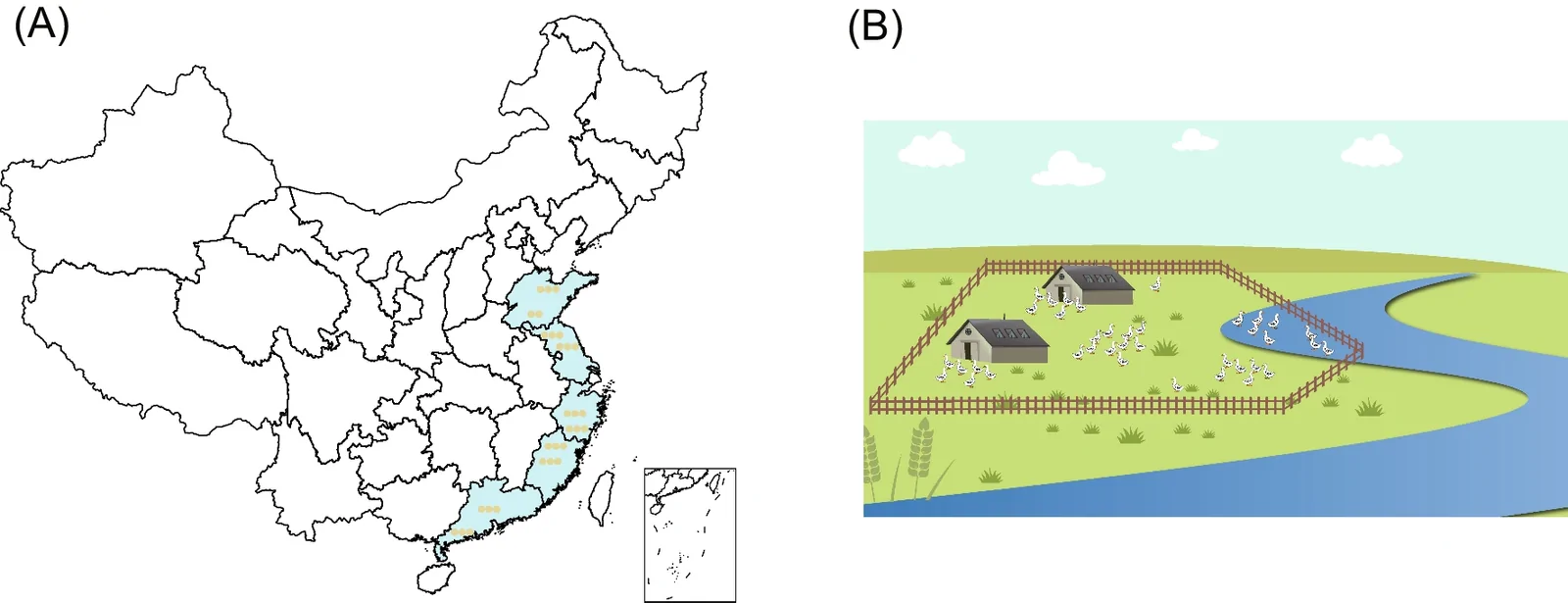
Schematic diagram of sample collection and farming patterns. **A** Samples were collected from 29 duck farms and surrounding environment in five southeastern coastal provinces of China. **B** Farming patterns in most of duck farms in southeastern coastal provinces of China

Feces and environmental samples were collected from 2018 to 2019 at 29 duck farms in Shandong, Jiangsu, Zhejiang, Fujian, and Guangdong, China, including 29 fecal, 32 soil, and 24 water samples (Fig. 1A). Fresh feces were placed in 10 mL sterile tubes. soil samples from each duck farm were collected using a five-point sampling method and were collected at depth 5 to 10 cm and transferred to sterile bags. Notably, the farms in Shandong province used an automatic water system and ducks are raised in cages with approximately 10 individuals per cage 1 m above the ground. In the other four provinces, ducks were fed and watered together houses of about 10,000 individuals and range freely in ponds and rivers (Fig. 1B). Therefore, the water samples in Jiangsu, Zhejiang, Fujian, and Guangdong Province were collected from uniformly selected sampling points near ponds and rivers in duck farms; water samples in Shandong Province are mainly collected in automatic water supply systems; and 500 mL water samples were collected from per sampling site and three to five sampling sites were selected per farm. All the farms were in use for at least 3 years and each produce > 10,000 commercial ducks annually. All samples were kept on dry ice for transportation to the laboratory and stored at − 80 °C before processing.

### DNA extraction

Samples collected at the same sampling site were mixed for metagenomic DNA extraction. For feces and soil samples, metagenomic DNA was extracted from approximately 0.25 g feces or soil using the QIAamp Power Fecal DNA isolation kit and DNeasy PowerSoil kits (Cat No 12830–50; Qiagen, Hilden, Germany) according to the manufacturer’s instructions. Water samples were filtered with sterile 0.45 μm filters, and metagenomic DNA was also extracted from the filter membranes using the DNeasy Power Water kit (Cat No 14900–50-NF, Qiagen). The quality, purity, and yield of the extracted DNA were quantified using 1% agarose gel electrophoresis and a NanoDrop2000 spectrophotometer (Thermo Fisher, Pittsburg, PA, USA), and Qubit 2.0 fluorometer (Thermo Fisher). The qualified DNA was stored at − 80 °C until further processing.

### Metagenomic sequencing

Metagenomic libraries were generated from 1 μg DNA per sample using the TruSeq DNA Sample Prep Kit (Illumina, San Diego, CA, USA) following the manufacturer’s recommendations. Briefly, DNA samples were sheared ultrasonically to 250–300 base pair fragments and end-polished, A-tailed, and ligated with the dual index adaptors for Illumina sequencing with further PCR amplification. All libraries were then sequenced using an Illumina Hiseq 2500 platform with 2 × 150 bp paired reads (Novogene, Beijing, China). Raw reads were quality trimmed by using Trimmomatic version 0.32 to remove paired reads with adapter, paired reads should be removed when the number of low-quality (Q ≤ 5) bases in single-ended sequencing reads exceeds 50% of the read length ratio.

### Analysis of ARGs

The relative abundance of ARGs was determined using the SARG 2.0 database (Yin et al. 2018). Putative ARG sequences were screened using USEARCH and normalized by the length. The sequence of extracted was annotated and classified using BLASTX with default parameters (the cutoff of e value of 10^−7^, sequence identity of 80%, and alignment length > 25 amino acids) (Feng et al. 2018). The relative abundance of ARGs was normalized with the ppm units (Zhang et al. 2020b).

### Analysis of ARG-carrying bacteria

Short reads in each sample were de novo assembled into contigs using MEGAHIT v1.1.3 with the default *k*-mer size (Li et al. 2015b). Open reading frames (ORFs) from each sample were searched against the ARG-OAP to identify the potential ARG-like ORFs using the BLASTP algorithm with the following parameters: sequence identity ≥ 80%, alignment coverage ≥ 70%, and e-value ≤ 1e − 5 (Xiong et al. 2018). Contigs harboring ARG-like ORFs were extracted using Prodigal software with the “meta” model (Hyatt et al. 2010). The taxonomy of the ARG-carrying bacterial communities was determined by annotating ARG-carrying contigs using Contig Annotation Tool, and the total reads were annotated using kraken2 with default parameters (von Meijenfeldt et al. 2019; Wood et al. 2019). The taxonomic list of the taxa at the species level was matched with a bacterial pathogen database (Li et al. 2015a). The mapped pathogens and the corresponding abundances were extracted for analysis.

### Statistical analysis and data visualization

ARG diversity analysis and principal coordinate analysis (PCoA) were measured using the “vegan” packages (Oksanen 2018). Venn diagrams were graphed by ImageGP (http://www.ehbio.com/ImageGP). The correlation analysis was diagrammed by “circlize” package (Gu et al. 2014). The heatmaps were visualized with the “pheatmap” package (Kolde 2019). The other figures were plotted by “ggplot2” and colored with “RColourBrewer” packages (Ginestet 2011; Neuwirth 2014).

### Data availability

All sequence data of 82 samples were deposited NCBI Sequence Read Archive (Accession No. PRJNA848116).

## Results

### Overall view of ARG in duck farms and surroundings environment

**Fig. 2:**
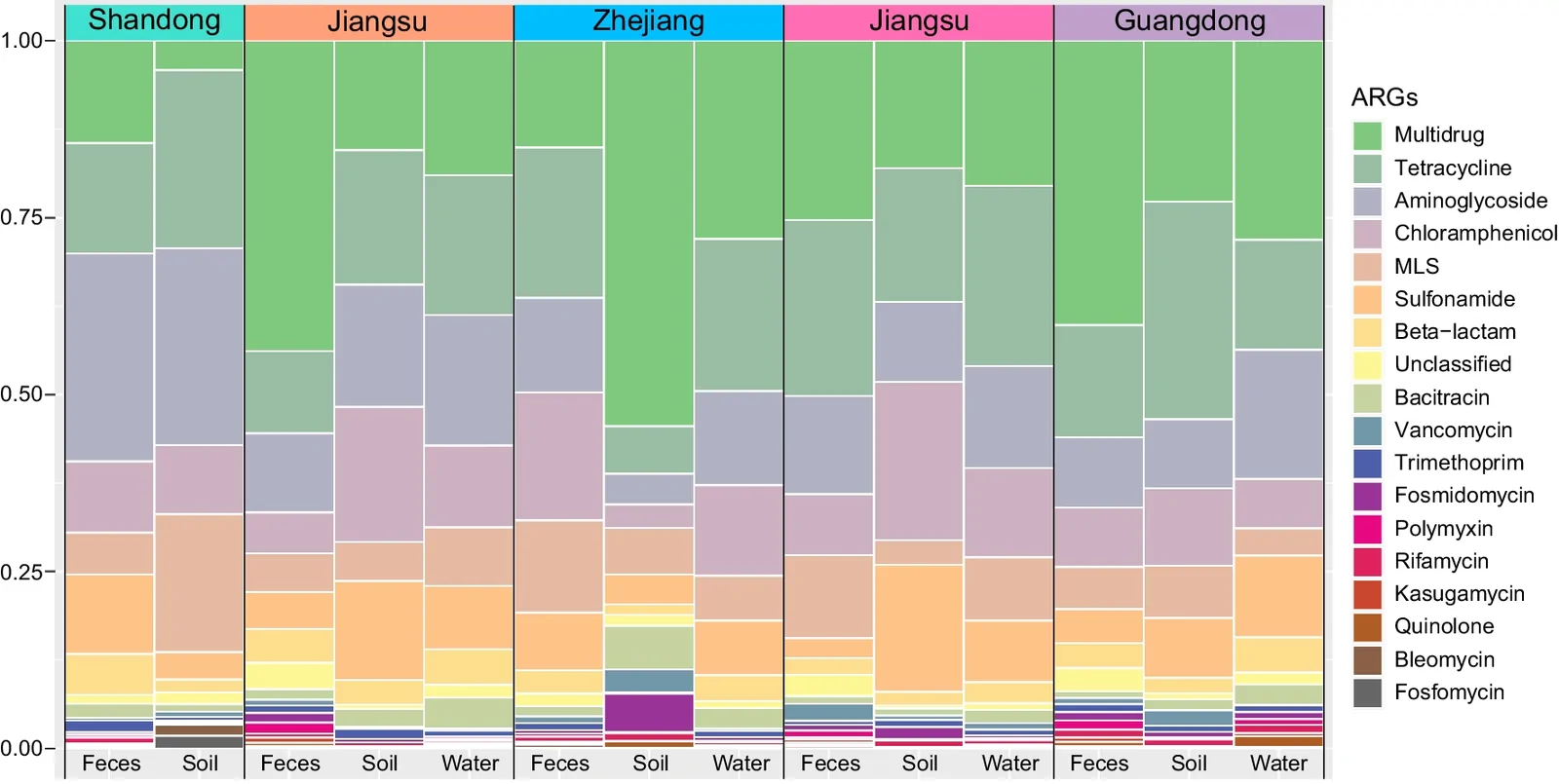
The rates of ARGs type prevalence across samples

A total of 82 metagenomic datasets were obtained from 85 samples collected from duck farms and surroundings environment, and DNA extraction failed from only thre samples. A total of 18 ARG types consisting of 823 subtypes from our 82 duck farms and surroundings environment samples. The six most dominant ARG types conferred resistance to multidrug (833 ± 1194 ppm) tetracyclines (574 ± 540 ppm), aminoglycosides (464 ± 507 ppm), chloramphenicols (323 ± 403 ppm), macrolide-lincosamide-streptogramins (MLS, 259 ± 290 ppm), and sulfonamides (209 ± 225 ppm). The sum of these six most dominant ARG types accounted for 58.4 ~ 94.7% of the total ARG relative abundance across the different samples (Fig. 2).

**Fig. 3:**
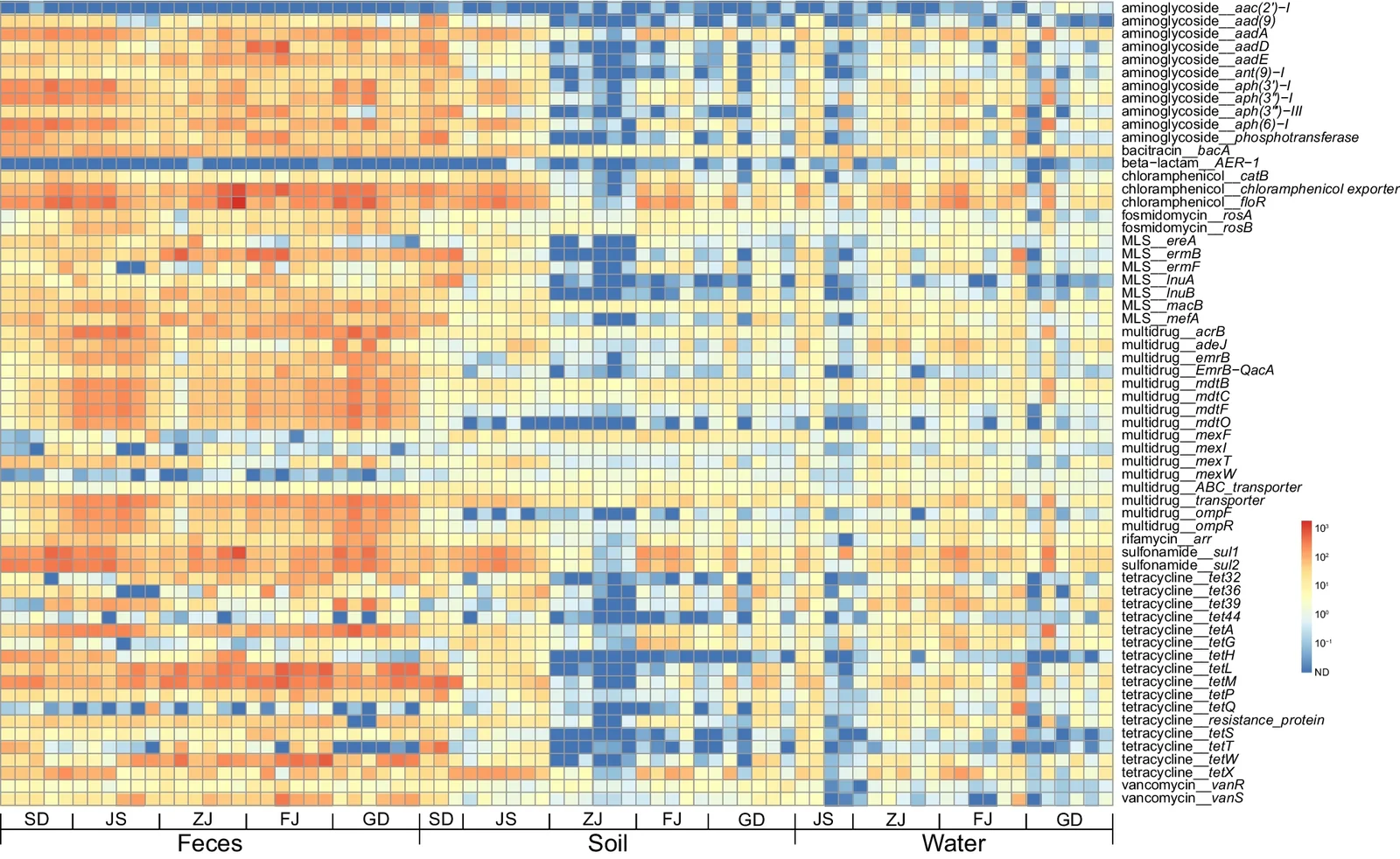
The 10 most abundant ARGs for each sample that totaled 62 subtypes for 82 metagenomic samples. In the main figure, each column represents one metagenome. Each row represents one ARG subtype. Abundance was transformed from the original abundance using the following formula: log_10_.^ARG relative abundance^ (unit: ppm)

Among these 823 subtypes, 529 subtypes encoded resistance to β-lactams followed by multidrug (*n* = 80), MLS (*n* = 48), aminoglycoside (*n* = 42), tetracycline (*n* = 44), and trimethoprim (*n* = 20) (Table S1). The 10 most abundant ARGs for each sample were extracted, and totally, 62 subtypes were shown in Fig. 3, with relative abundance levels of 56.54 ~ 94.16% of the corresponding total ARG relative abundance. The gene *floR* was the most abundant subtype encountered (6.91%, 159 ± 238 ppm) in all samples, followed by chloramphenicol exporters (5.49%, 126 ± 156 ppm), *sul*1 (5.14%, 118 ± 138 ppm), *tet*M (5.00%, 115 ± 133 ppm), *sul*2 (4.30%, 99 ± 104 ppm), and *tet*L (3.75%, 86 ± 139 ppm) (Table S2 and Table S3).

### Comparison of ARG relative abundance among the different samples

**Fig. 4:**
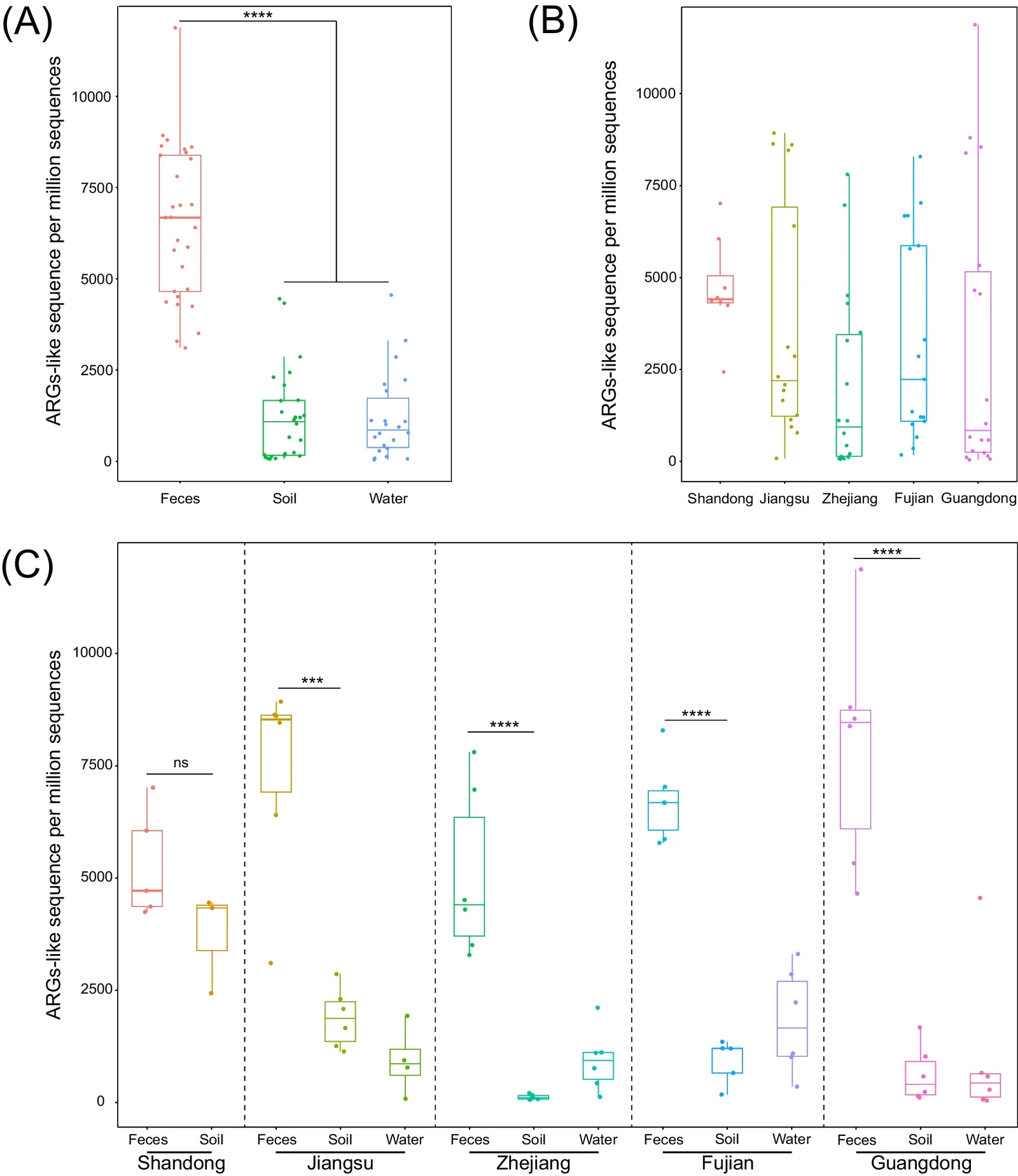
Comparison of total ARG relative abundance across samples. **A** Sample type, **B** province, and **C** between origin in the same province. Boxes denote the interquartile (IQR) between the first and third quartiles (25th and 75th percentiles, respectively) and the line inside denotes the median. Whiskers denote the lowest and highest values within 1.5 times and the IQR from the first and third quartiles, respectively. (ns, *P* > 0.05; ***, *P* < 0.001; ****, *P* < 0.0001)

At the type level, relative ARG abundance in fecal sample (6512 ± 2074 ppm) was significantly higher than soil and water samples (1202 ± 1152 ppm and 1217 ± 1226 ppm; *P* < 0.0001) (Fig. 4A). ARG relative abundance could also be ranked by province as from high to low were Shandong (4702 ± 1267 ppm), Jiangsu (3700 ± 3166 ppm), Fujian (3282 ± 2699 ppm), Guangdong (3199 ± 3755 ppm), and Zhejiang (2042 ± 2405 ppm) (Fig. 4B). There was no significant difference for ARG relative abundance between soil and fecal samples in Shandong (*P* > 0.05), while ARG relative abundance in fecal is significantly higher than soil in other provinces (*P* < 0.001) (Fig. 4C). These data indicated that duck feces are the reservoirs of ARGs.

We identified six dominant ARG types in our sample collection, and multidrug, tetracycline, aminoglycoside, chloramphenicol, MLS, and sulfonamide ARGs were more abundant in fecal samples compared with soil and water samples. In the latter, the dominant ARG types were significantly higher in soil compared with water (*P* < 0.05), except multidrug (Table S4). In addition, the relative abundance of tetracycline, aminoglycoside, chloramphenicol, MLS, and sulfonamide resistance genes in all samples from Shandong were more abundant than other provinces. However, multidrug ARGs in all samples from Jiangsu and Guangdong were more abundant than Shandong, Zhjiang and Fujian (Table S5).

At the subtype level, the relative abundance of dominant ARG subtypes in fecal samples exceeded those in soil and water samples, although 6 subtypes (*floR*, chloramphenicol exporter, *tet*M, *sul2*, *aad*A, *erm*B) were enriched in soil samples compared with water. In contrast, *sul1*, multidrug transporter, *aph*(6)-I, *aph*(3″)-I, and *tet*A were lower in the soil samples (Table S2). The six dominant ARG subtypes (*floR*, chloramphenicol exporter, *sul1*, *tet*M and *sul2*) were more abundant in Shandong samples, while *tet*L in Fujian samples exceeded those from the other provinces (Table S3).

### Comparison of ARG composition

**Fig. 5:**
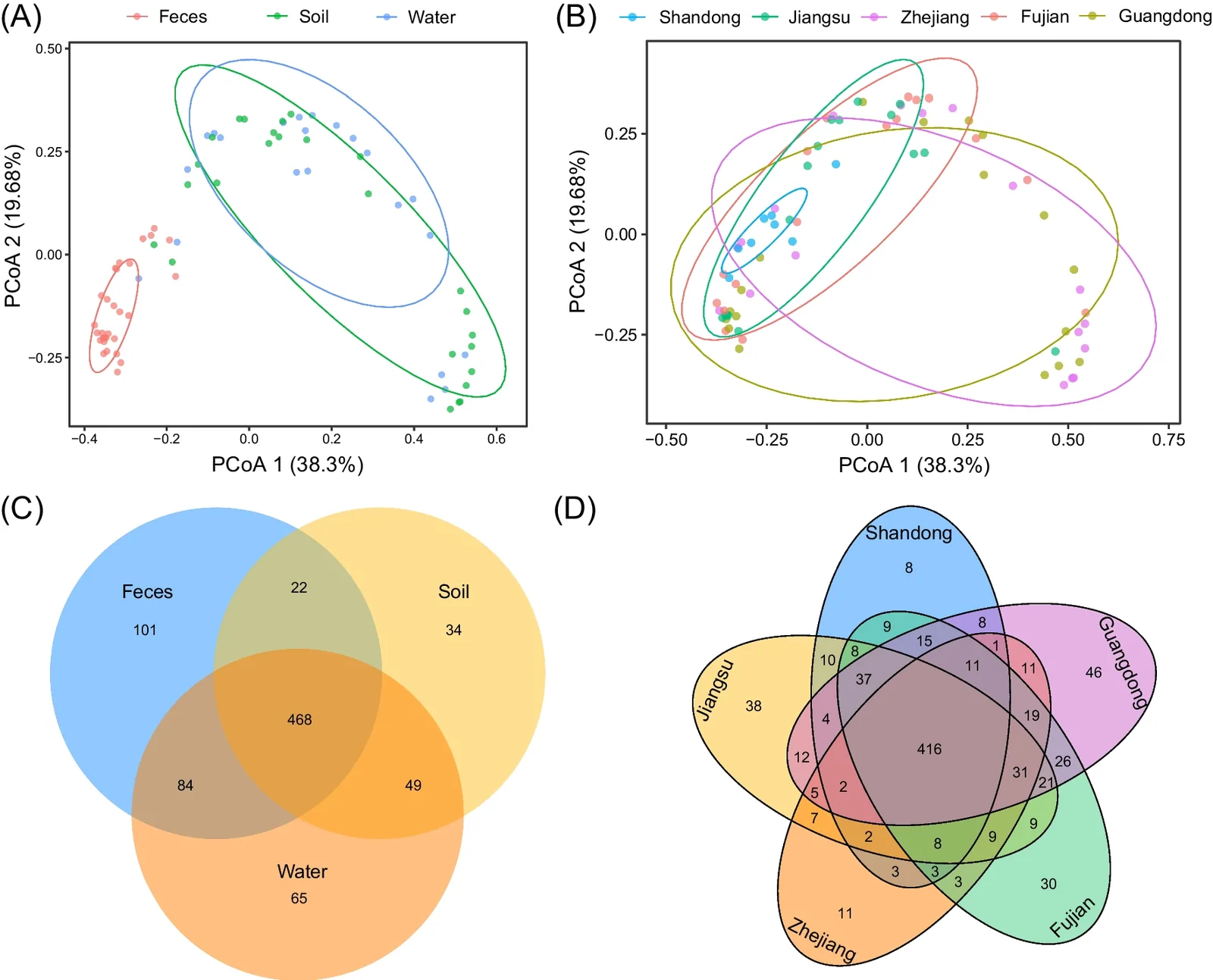
Composition similarity and shared and unique ARG subtypes among different samples. ARG subtype compositions **A** for sample type and **B** by province. Numbers of shared and unique ARG subtypes by **C** sample type and **D** province

A similarity analysis of ARG subtypes composition among 77 samples was performed using PCoA. In particular, the first two PCoA components of Bray–Curtis distance explained a high proportion of variance (57.98%) and the composition of ARGs subtypes were similar between water samples and soil samples and significantly different from that of the fecal samples. The ARG subtypes in feces were also closely clustered (Fig. 5A). Moreover, although the composition of ARGs subtypes were similar between samples from five provinces, the samples from Shandong were closely clustered and samples from Zhejiang, Fujian, and Guangdong possessed a level of variance (Fig. 5B).

A calculation of shared and unique ARG subtypes indicated that 468/823 ARGs were shared by fecal, soil, and water samples. The fecal (*n* = 675) and water (*n* = 666) samples harbored exceeded that of the soil samples (*n* = 573) (Fig. 5C). In addition, the numbers of ARGs subtypes could be ranked as Guangdong (*n* = 665), Fujian (*n* = 655), Jiangsu (*n* = 619), Shandong (*n* = 545), and Zhejiang (*n* = 542) (Fig. 5D), indicating more diverse resistomes in samples from Guangdong, Fujian, and Jiangsu. In addition, the most abundant ARG types were multidrug resistance genes both in fecal (30.2%) and water samples (23.7%), while tetracycline resistance gene was the dominant ARG type in soil samples, accounting for 22.3% (Fig. S1).

### ARG bacterial hosts

A total of 8567 ARG-carrying contigs were obtained from 77 samples through metagenomic assembly and aligned to the structured ARG database. Within this group, 25.7% (2205/8567) of the contigs were taxonomically assigned to eight phyla that were dominated by the *Proteobacteria* (1188/2205, 53.9%), *Firmicutes* (756/2205, 34.3%), *Bacteroidetes* (136/2205, 6.2%), and *Actinobacteria* (118/2205, 5.4%). The dominant bacterial phyla were *Proteobacteria* and *Firmicutes* in all of the samples, while *Actinobacteria* were higher than *Bacteroidetes* in the soil and water samples (Table S6).

**Fig. 6:**
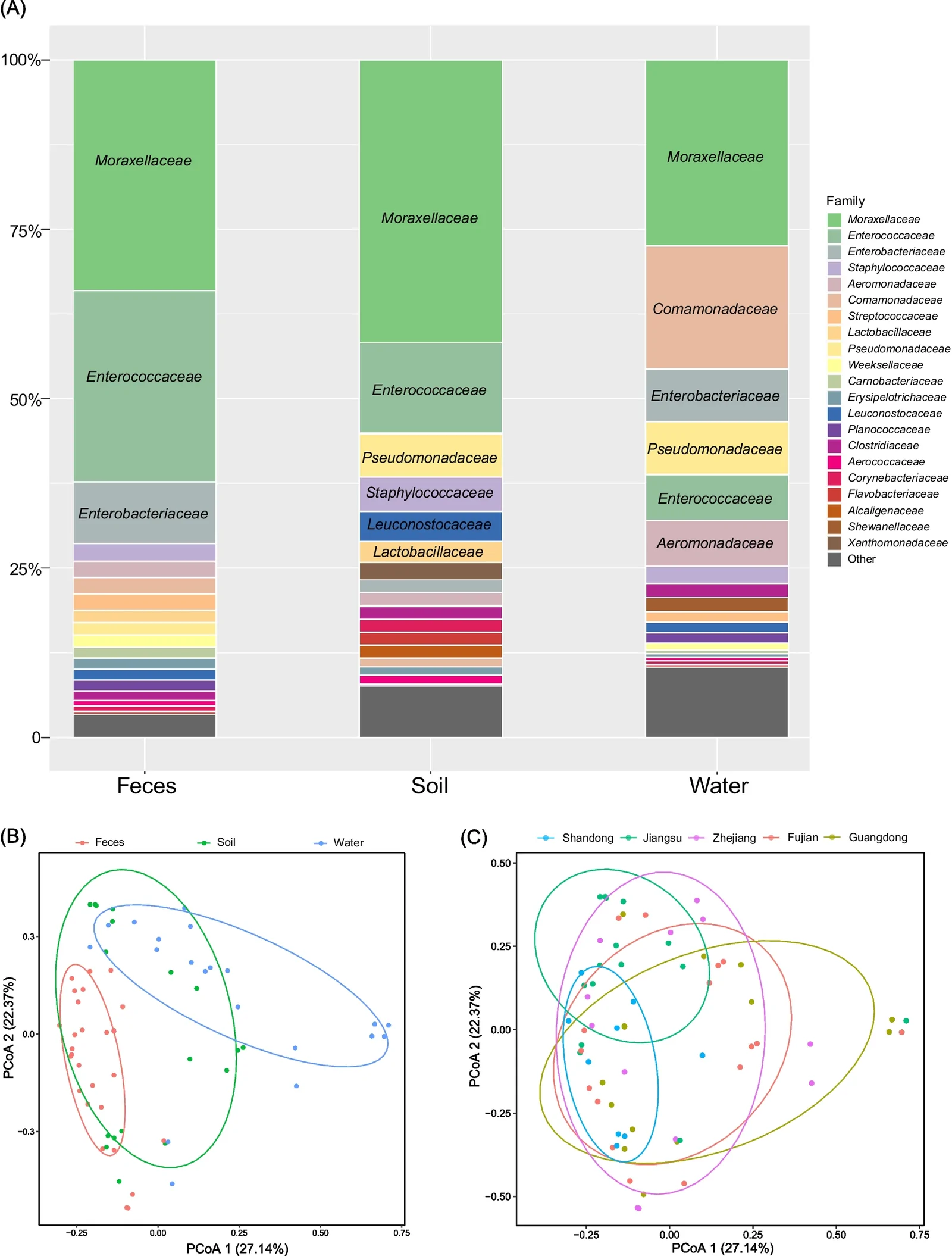
Comparisons of ARG bacterial hosts in feces, soil, and water. **A** ARG composition by sample type. **B** Similarity of ARG-carrying host compositions among sample types. **C** Similarity of ARG-carrying host compositions for Shandong, Jiangsu, Zhejiang, Fujian, and Guangdong

In addition, only 9.9% (847/8567) contigs were classified into 41 bacterial families that were primarily composed of *Moraxellaceae* (228/847, 34.0%), *Enterococcaceae* (174/847, 20.5%), *Enterobacteriaceae* (63/847, 7.4%), *Comamonadaceae* (49/847, 5.8%), and *Pseudomonadaceae* (34/847, 4.0%) (Fig. S2 and Table S7). Notably, vancomycin ARGs were primarily harbored by *Enterococcaceae* (Fig. S2A). In contrast, the *bac*A resistance gene possessed the greatest host diversity (Fig. S2B). Fecal, soil, and water samples possessed 496, 158, and 193 contigs that could be aligned into the 27, 28, and 26 ARG-carrying bacterial families, respectively, and 16 families were shared across the 3 sample types (Fig. S3A). The numbers of ARG-carrying bacterial families were as follows 29 from Fujian, 25 from Shandong, 22 from Zhejiang, 20 from Guangdong, and 28 from Zhejiang, and included eight presented in all provinces (Fig. S3B). These distinctions were further defined and *Moraxellaceae* predominated in all three sample types, while *Comamonadaceae* levels in water exceeded that of soil and fecal samples and the *Pseudomonadaceae* equally represented in water samples and soil samples. In addition, the proportion of ARGs carried by *Moraxellaceae* was highest in Shandong, Jiangsu, and Zhejiang samples, while ARGs carried by *Enterococcaceae* were highest Fujian and Guangdong samples (Fig. 6A). ARGs distributed in *Staphylococcaceae* to a lesser degree although a higher percentage was detected in samples from Shandong (Fig. S4).

A similarity analysis of ARG-carrying bacterial family composition among 77 samples was also conducted, and PCoA revealed that the composition of ARG-carrying bacterial families in soil was similar to fecal and water samples, although fecal and water samples differed significantly (Fig. 6B). Furthermore, the composition of ARG-carrying bacterial families in samples from Shandong was closely clustered, whereas samples from Zhejiang, Fujian, and Guangdong exhibited evident scattering (Fig. 6C).

#### Characterization of ARG-carrying HPB

In this study, only 2.5% (212/8567) of the contigs could be classified and these included 59 bacterial species. Of which, the highest proportions were found for *Enterococcus cecorum* (74/212, 34.91%), *Enterococcus faecium* (14/212, 6.6%), *Erysipelotrichaceae* bacterium MTC7 (11/212, 5.2%), *Acinetobacter baumannii* (9/212, 4.2%), and *Acinetobacter calcoaceticus* (9/212, 4.2%) (Table S8). A total of 10 ARG-carrying human pathogenic bacteria (HPB) were detected in all samples though compared to bacterial pathogen database which contains 538 pathogenic species and distributed in 41 samples. In addition, *Enterococcus faecium*, *Acinetobacter baumannii*, and *Staphylococcus aureus* were detected in 14 (14/41, 34.1%), 9 (9/41, 22.0%), and 5 (5/41, 12.2%) samples, respectively. These numbers of ARGs-carrying HPB were twofold and fivefold higher in fecal compared with soil and water samples, respectively (Table S9).

Correlations between pathogenic bacteria, ARGs subtypes, and location of sample indicated that *E. faecium* were most likely to harbor the chloramphenicol resistance gene *cat* and distributed across multiple provinces. All *A. baumannii* carried the trimethoprim resistance gene *dfrA20* and predominated in Jiangsu provinces. The *Staphylococcus aureus* mainly carried MLS resistance gene *vga*E, and detected in Shandong and Fujian provinces. It is important to mention that Shandong province have been severely contaminated by a wide variety of ARGs-carrying HPB (Fig. S5).

## Discussion

### Distribution characteristics of ARGs

In this study, metagenomic analysis was used to detect the diversity and abundance of ARGs in duck farms and their surrounding environment. A total of 18 ARG types were identified in all the samples and dominated by multidrug, tetracycline, aminoglycoside, chloramphenicol, MLS, and sulfonamide resistant genes. These six were identical in ranking to those found on pig farms excepting that multidrug resistance genes were higher for duck farms aminoglycoside and sulfonamide resistance genes were lower than pig farms (Zhang et al. 2021). In addition, the relative abundance of ARGs in fecal samples was higher than for water and soil samples, which may pose a potential pollution threat to the surrounding environment. Meanwhile, ARGs have the potential to contaminate surrounding underground drinking water supplies via pig farm wastewater (He et al. 2016). The relative abundance of ARGs for pig farms were highest in Guangdong and Heilongjiang relative to Sichuan and Hebei (Wang et al. 2019). In contrast, we found that Guangdong levels were the lowest and Shandong the highest. In addition, ARG-carrying bacteria were highly endemic in backyard animals, commercial broiler farms, surrounding environment, and in the food chain in Shandong (Li et al. 2019; Wang et al. 2017). Collectively, these studies indicated that food animals in Shandong province may be serving as an enormous ARG reservoir. This indicates that farms are generally contaminated by ARGs and it is necessary to conduct continuous monitoring of ARGs in food-borne animals in Shandong Province and other provinces of China.

A total of 823 ARG subtypes were detected in this study, and among the 62 most prevalent ARG subtypes, *floR*, chloramphenicol exporter, *sul1*, *tetM*, *sul2*, and *tetL* were the most common. A total of 257 ARG subtypes had been previously detected in pig farms, and *tet(W)*, *tet(Q)*, *tet(44)*, *tet(37)*, and *tet(40)* were the predominant resistance gene (Joyce et al. 2019). These results suggest that the prevalence of sulfonamides and tetracycline resistance genes in duck and pig farms may be associated with the high frequency of use of these two drugs and sulfonamides and tetracycline drugs should be used reasonably in livestock and poultry breeding. Furthermore, ARG composition was more diverse in broiler chicken farms compared with the pig farms (Munk et al. 2018). In addition, predominant ARG subtypes in fecal samples were higher than environmental samples. PCoA indicated that composition of ARGs in soil and water were similar and only distantly related to the fecal samples. Therefore, the distribution of ARG subtypes may be primarily influenced by sample types (Mencia-Ares et al. 2020). It is also noteworthy that the relative abundance of *flo*R was the highest in Zhejiang, Jiangsu, and Guangdong and the highest diversity was found in Guangdong and Fujian. These results were similar to prior studies on different pig farms in European countries that indicated a stark difference in diversity and relative abundance in different fecal samples (Munk et al. 2018). Overall, the diversity and relative abundance of ARGs may be influenced by geography.

### Bacterial hosts

Bacterial communities are key factors shaping ARG prevalence. However, there is not a clear co-occurrence pattern of ARG subtypes and specific bacteria and the assembly of long ARG-carrying contigs is required for a more accurate taxonomic classification of ARGs (Zhang et al. 2019). Therefore, ARG-carrying contigs were extracted and *Proteobacteria*, *Firmicutes*, *Bacteroidetes*, and *Actinobacteria* were identified as the major ARG hosts; the results in duck farms were similar to swine, chicken, and wild animals. For instance, *Proteobacteria* and *Firmicutes* predominated swine manure (Wang et al. 2021a; Zhang et al. 2021), while in dairy cow manure, ARGs originated from *Proteobacteria*, *Firmicutes*, *Bacteroidetes*, and *Actinobacteria* (Wichmann et al. 2014). *Proteobacteria* was the predominant bacterial host and harbored the most diverse ARGs in broiler chickens and migratory birds (Cao et al. 2020).

We identified seven bacterial families (*Moraxellaceae, Enterococcaceae*, *Enterobacteriaceae*, *Comamonadaceae*, *Pseudomonadaceae*, *Aeromonadaceae*, and *Staphylococcaceae* as major potential ARG hosts. The *Moraxellaceae* hosted multidrug and bacitracin resistance genes including *bacA*, *mexT*, *adeJ*, *adeK*, and *abaQ*, although multidrug, MLS, and aminoglycoside resistance genes dominated for this family in swine manure (Zhang et al. 2021). *Moraxellaceae* were primarily represented by *Acinetobacter* that was the most prevalent ARG host (Zhang et al. 2020a). *Acinetobacter* were the predominated sulfonamide resistant bacteria in wastewater and shrimp ponds (Phuong Hoa et al. 2008). Multidrug resistance genes were carried by *Enterobacteriaceae*, and bacitracin resistance genes were carried by *Enterococcaceae* and *Comamonadaceae*. In the giant panda intestine, *Enterobacteriaceae* have been previously documented as the primary contributors to multidrug and tetracycline resistant genes and *Enterococcaceae* were associated with MLS carriage (Hu et al. 2020). The *Burkholderiaceae* were more prevalent in increasing multidrug resistance genes abundance in activated sludge reactors treated antibiotic production wastewater (Zhao et al. 2020). It is noteworthy that the multidrug resistance gene *bac*A possessed the greatest host diversity for the duck farms and surrounding environment. In contrast, *Comamonadaceae* were assigned as the major potential hosts of *bac*A in swine manure, wastewater, and soil (Zhang et al. 2021). In addition, the frequent hosts of *bac*A were *Aeromonadaceae* and *Fusobacteria* in a full-scale drinking water treatment plant (Jia et al. 2020). This is evidenced by our data which show that the potential bacterial hosts of ARGs are diverse, increasing the risk of transmission of ARGs between different bacterial species. In addition, this investigation also provided a scientific basis for taking measures to prevent the spread of resistance in farms.

### ARG-carrying human pathogenic bacteria

Bacterial pathogens are the major concerns regarding public health, and we found ten ARG-carrying human pathogens, which included *E. faecium*, *A. baumannii*, and *S. aureus* that are members of the antimicrobial-resistant ESKAPE pathogens that also include *E. faecium*, *S. aureus*, *K. pneumoniae*, *A. baumannii*, *P. aeruginosa*, and *Enterobacter* spp (De Oliveira et al. 2020). These ESKAPE pathogens have reduced treatment options for serious infections and increased the burden of disease and death (De Oliveira et al. 2020). Our previous study obtained a small number of carbapenem-resistant *K. pneumoniae* and *P. aeruginosa* within the samples of the same batch (Wang et al. 2021c). Coincidentally, several zoonotic food-borne pathogens including *Salmonella* and *Clostridium perfringens* have also been detected on duck farms in China (Chen et al. 2020; Liu et al. 2022; Xiu et al. 2020). Thus, continued vigilance for ESKAPE pathogens in food animals is necessary especially since fecal serve as a reservoir for contamination of the surrounding environment (Yang et al. 2020).

Correlation analysis for human pathogenic bacteria and ARGs indicated that *E. faecium* was linked to chloramphenicol resistance genes, *A. baumannii* and *S. aureus* to sulfonamide and MLS resistance genes, respectively. *E. faecium* was also to harbor resistance to vancomycin (*vanA*, *B*, *C*, *D*, *G*, *L*, *M*, *N*, *P*), phenicol-oxazolidinone (*poxtA*), and linezolid (*optrA*) (Cattoir and Giard 2014). ARG analysis of *A. baumannii* genomes in public databases indicted that almost identical resistance genes for all isolates; *armA*, *aph(3′)-VI-a*, *aph(6′)-Id*, and *strA* genes all conferring resistance to aminoglycosides, *tetB* gene encoding for tetracycline resistance, *mphE* and *mrsE* genes, both responsible for macrolide resistance and *sul1* and *sul2* genes for sulphonamide resistance (Jia et al. 2019). Correspondingly, *S. aureus* harbored multiple ARGs collectively conferring resistance to aminoglycosides (*aph(6)-Id*, *aph(3′)-III*), β-lactam (*blaZ*, *mecA*), chloramphenicol (*fexA*), fosfomycin (*fosB*), lincosamide (*lnuA*), MLS (*ermB*, *ermC*), tetracyclines (*tetL*, *tetM tetK*), and trimethoprim (*dfrA1*, *dfrG*) (Gu et al. 2020). Importantly, the *E. faecium* and *S. aureus* harbored multiple ARGs that were widely distributed across retail samples of raw beef, sheep, and lamb meat, and were found in urban settings and can cause life-threatening blood stream infections due to limited treatment options (Gu et al. 2020; Pınar 2021; Wang et al. 2021d). Human pathogenic bacteria infections remain among the major worldwide causes of morbidity and mortality, even more worryingly, the emergence and transmission of ARG-carrying human pathogenic bacteria posed a growing global threat to human health. Therefore, to arrest the dissemination of resistant human pathogenic bacteria, these farms should develop more rational rules for antibiotic use.

This study has several limitations. First, this investigation neglected longitudinal monitoring to examine ARG persistence with their bacterial hosts. Second, antibiotic residue detection was lacking in this study. Therefore, the association between the antibiotic residues and ARG diversity requires further investigation. Third, environmental samples were not comprehensive enough and farm worker, drinking water, dust, flies, and aerosol samples were not collected. Therefore, the potential exposure risks of ARGs in duck farms still needs to be further studied.

In conclusion, a total of 18 ARG types consisting of 823 subtypes were detected in the duck farms and surrounding environment and the six dominant ARG types conferred resistance to multidrug, tetracycline, aminoglycoside, chloramphenicol, MLS, and sulfonamide. Among these 823 subtypes, *floR* was the most abundant in all samples followed by chloramphenicol exporter, *sul1*, *tetM*, *sul2*, and *tetL*. At the type level, the relative abundance of ARGs in feceal samples was significantly higher than soil and water samples. PCoA revealed that the composition of ARG subtypes were similar between water and soil samples and significantly difference from fecal samples. However, the composition of ARG subtypes were similar between samples from all five provinces. Bacterial ARG hosts were taxonomically assigned to eight phyla that were dominated by the *Proteobacteria*, *Firmicutes*, *Bacteroidetes*, and *Actinobacteria*, and 41 bacterial families were deduced as the potential ARG hosts and *Moraxellaceae*, *Enterococcaceae*, *Enterobacteriaceae*, *Comamonadaceae*, and *Pseudomonadaceae* carried more diverse ARG subtypes than other families. In addition, some bacterial pathogens could be enriched in duck feces and could serve as ARG carriers. This study provides a comprehensive overview of the diversity and abundance of ARGs in duck farms and highlights the possible role of duck feces as ARG disseminators into the environment.

## Supplementary information

Below is the link to the electronic supplementary material.

Supplementary file1 (PDF 5709 KB)

## Data Availability

All data generated or analyzed during this study are included in this article.
