# Supplementary material for: Distribution of antibiotic resistance genes and their pathogen hosts in duck farm environments in south-east coastal China
Source: Appl Microbiol Biotechnol. 2024 Jan 15;108:136. doi: 10.1007/s00253-023-12842-4 (PMC10789667; doi:10.1007/s00253-023-12842-4)
Supplement: Supplementary file 1 [file 253_2023_12842_MOESM1_ESM.pdf]

Distribution of antibiotic resistance genes and their pathogens hosts in duck farm environments in south-east coastal China

Kaidi Liu [a,b,c,d]†, Minge Wang [a,b,c,d]†, Yin Zhang [a,c,d], Chang Fang [a,c,d], Rongmin Zhang [a,c,d], Liangxing Fang [a,c,d], Jian Sun [a,c,d,e], Yahong Liu [a,c,d,e,f], Xiaoping Liao [a,c,d]*

[a] National Risk Assessment Laboratory for Antimicrobial Resistance of Animal Original Bacteria, South China Agricultural University, Guangzhou, P. R. China.

[b] School of Agricultural Science and Engineering, Liaocheng University, No.1 Hunan Road, 252000, Liaocheng, Shandong, China.

[c] Laboratory of Veterinary Pharmacology, College of Veterinary Medicine, South China Agricultural University, Guangzhou 510642, P. R. China.

[d] Guangdong Provincial Key Laboratory of Veterinary Pharmaceutics Development and Safety Evaluation, South China Agricultural University, Guangzhou 510642, China.

[e] Guangdong Laboratory for Lingnan Modern Agriculture, Guangzhou, 510642, P. R. China.

[f] Jiangsu Co-Innovation Center for the Prevention and Control of Important Animal Infectious Diseases and Zoonoses, Yangzhou University, Yangzhou 225009, P. R. China.

* Address correspondence to Xiao-Ping Liao, E-mail: xpliao@scau.edu.cn.

†These two authors contributed equally to this work.

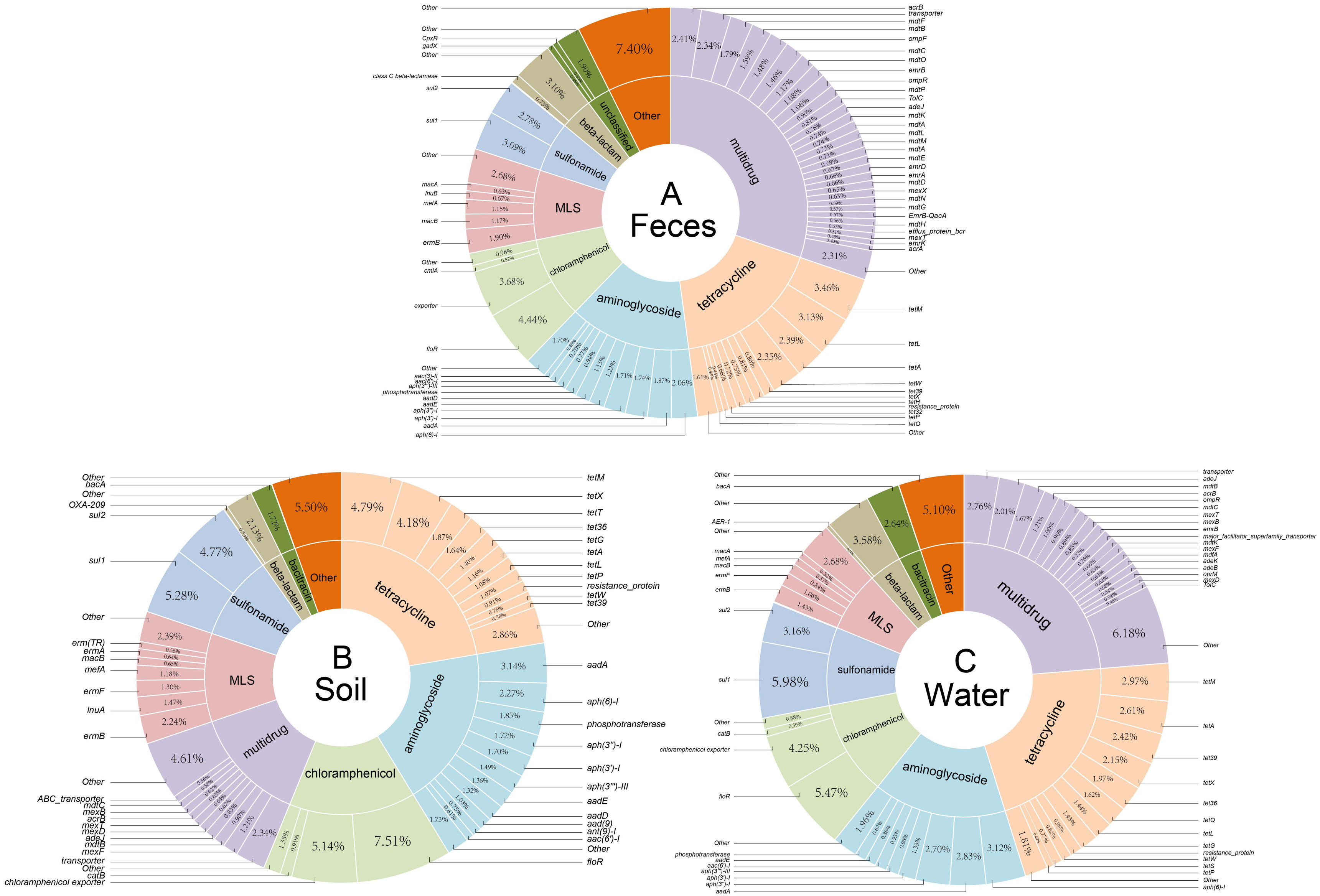

Fig S1. Doughnut charts showing the details of ARGs composition in (A) the feces samples, (b) the soil samples and (c) the water samples.

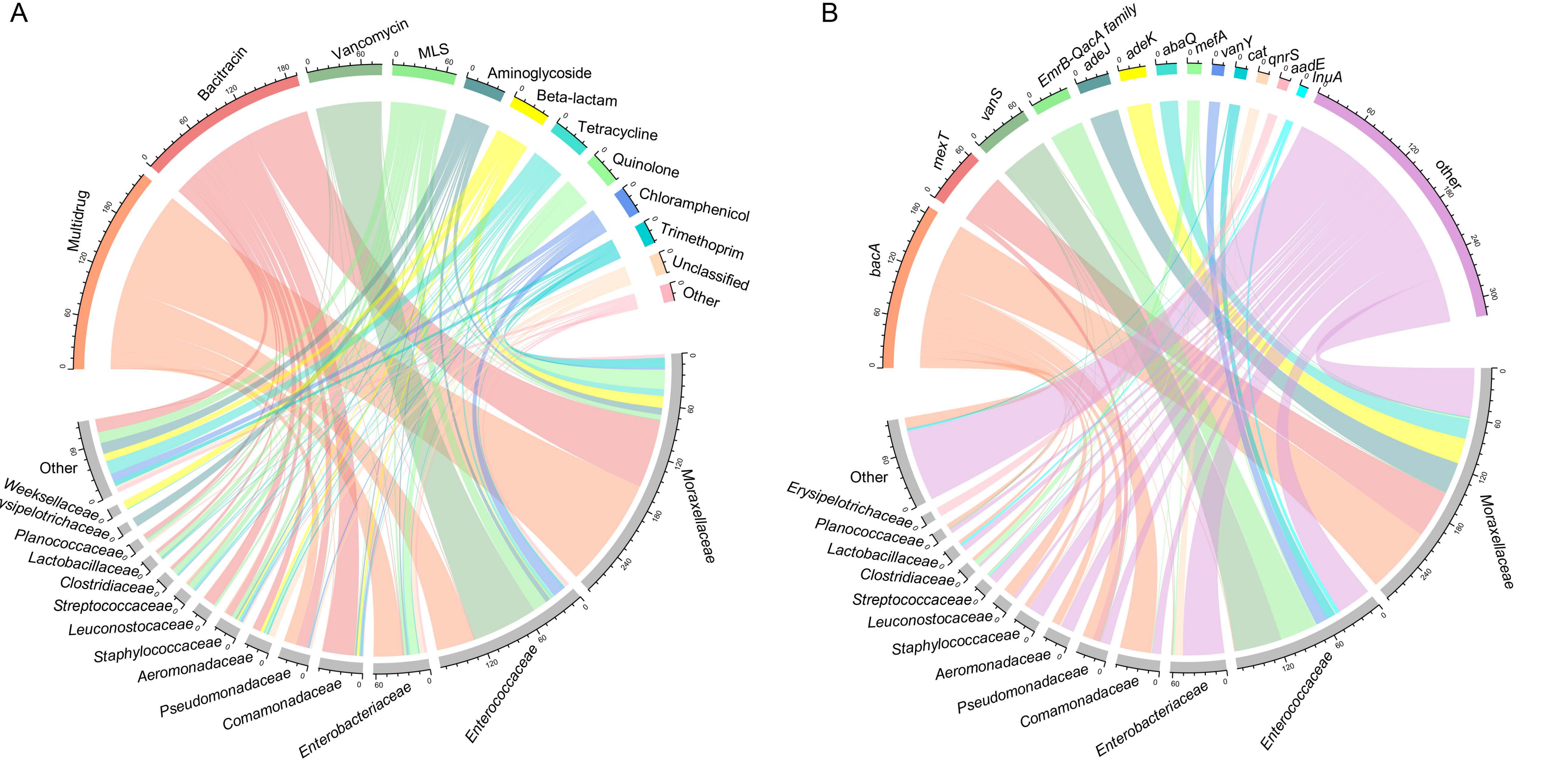

Fig S2. Correlations between ARGs/ARGs subtype and their bacterial hosts. (A) Analysis orrelations between ARGs and their bacterial hosts at the family level. (B) Analysis correlations between the predominant ARGs subtype and their bacterial hosts at the family.

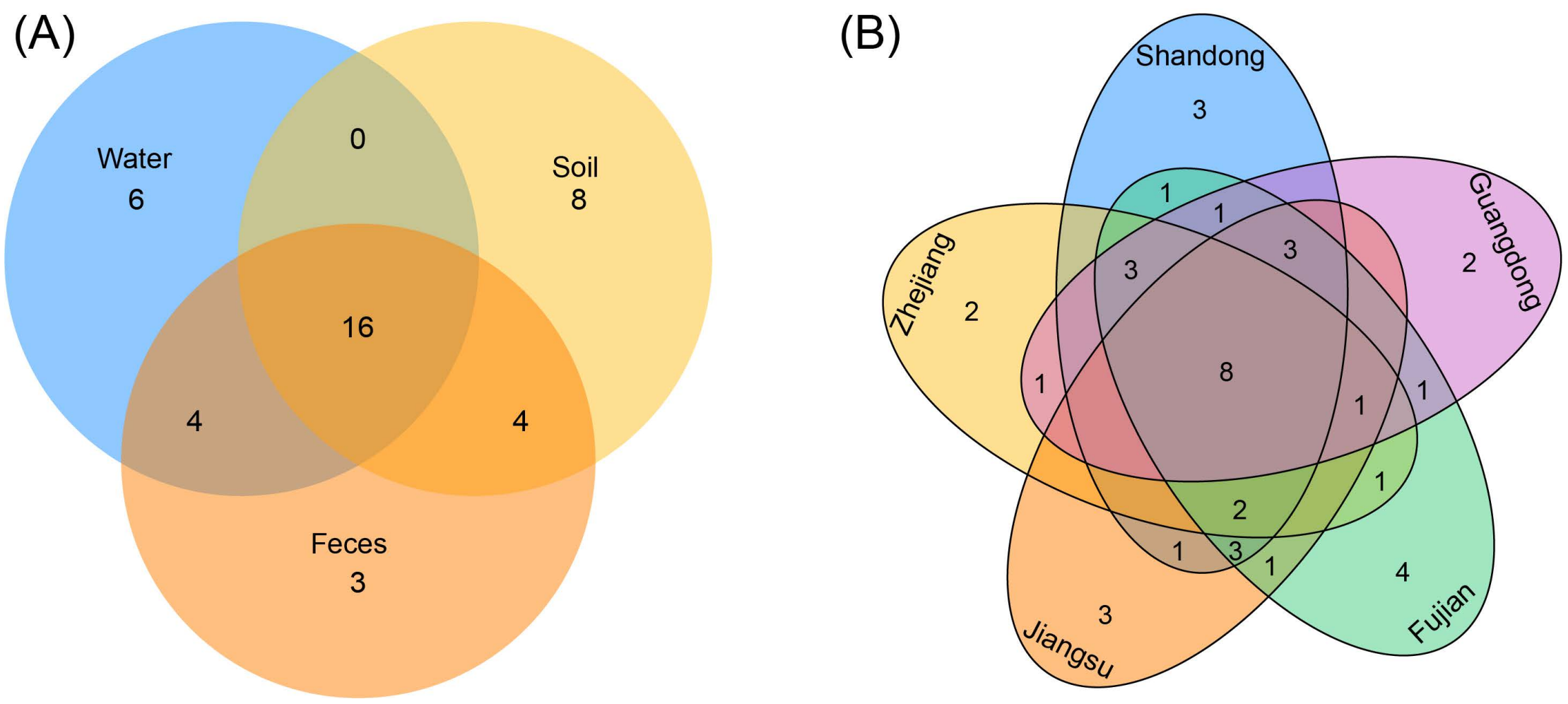

Fig S3. Venn diagram showing the numbers of shared and unique ARGs subtype. (A) the numbers of shared and unique ARGs subtype among the feces, soil and water. (B) the numbers of shared and unique ARGs subtype among the Shandong, Jiangsu, Zhejiang, Fujian and Guangdong

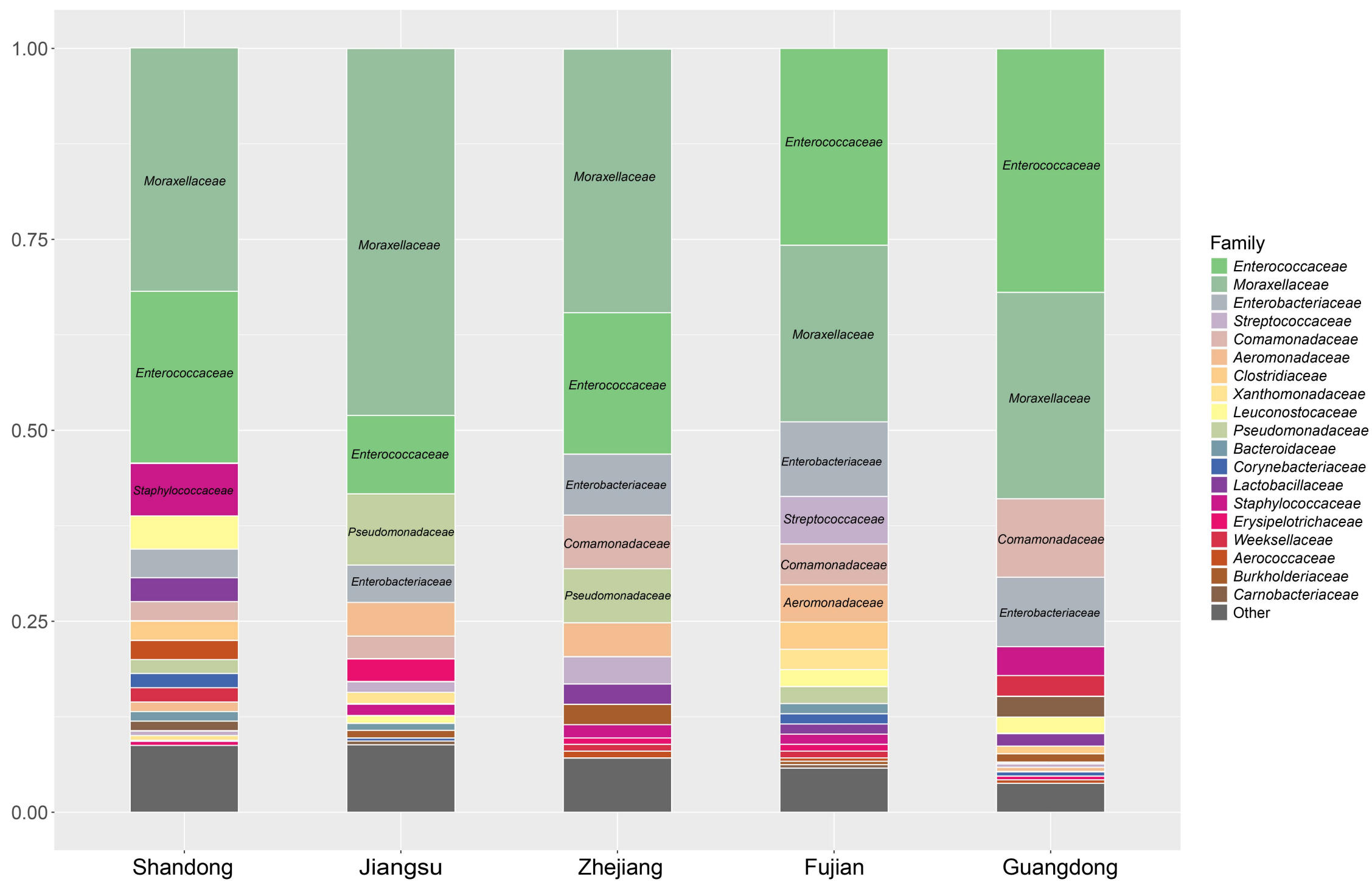

Fig S4. Composition of ARG bacterial hosts the Shandong, Jiangsu, Zhejiang, Fujian and Guangdong

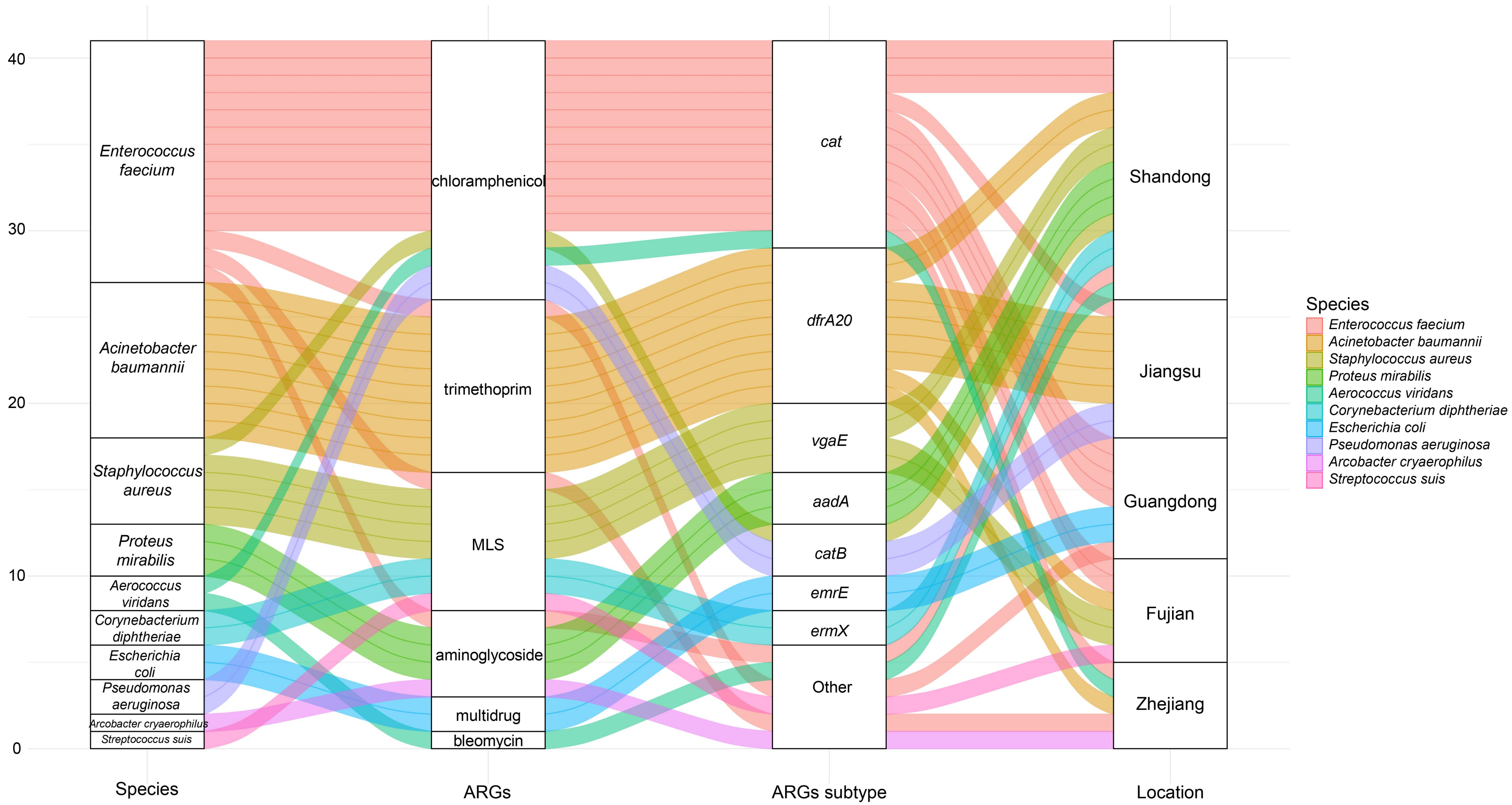

Fig S5. Correlations between human pathogenic bacteria, ARGs, ARGs subtype and location.

Table S1. The numbers of ARGs subtype in each ARGs type

Types of ARGs	Numbers of subtype	Types of ARGs	Numbers of subtype
beta-lactam	529	quinolone	6
multidrug	82	fosfomycin	3
MLS	48	rifamycin	3
aminoglycoside	42	sulfonamide	2
tetracycline	36	bacitracin	2
trimethoprim	20	bleomycin	2
vancomycin	17	fosmidomycin	2
unclassified	15	kasugamycin	1
chloramphenicol	13	polymyxin	1

Table S2. The average relative abundance of ARG subtypes in feces, soil and water, respectively

ARG Subtypes	Feces (ppm)	Soil (ppm)	Water (ppm)	Total (ppm)
floR	289 ± 335	91 ±91	66 ± 70	159 ± 238
chloramphenicol exporter	239 ± 194	63 ± 69	51 ± 54	126 ± 156
*sul*1	201 ± 176	64 ± 64	72 ± 80	118 ± 138
*tet*M	225 ± 114	58 ± 114	36 ± 54	115 ± 133
*sul*2	181 ± 111	58 ± 66	38 ± 45	99 ± 104
*tet*L	204 ± 165	13 ± 20	17 ± 43	86 ± 139
multidrug transporter	152 ± 85	29 ± 20	33 ± 33	76 ± 81
*tet*A	156 ± 115	14 ± 13	31 ± 71	72 ± 104
aph(6)-I	134 ± 104	28 ± 40	38 ± 58	71 ± 90
*aad*A	122 ± 71	38 ± 38	34 ± 37	69 ± 67
*acr*B	157 ± 123	8 ± 4	15 ± 24	66 ± 104
*tet*W	153 ± 152	9 ± 15	10 ± 11	64 ± 117
*erm*B	124 ± 146	27 ± 74	17 ± 54	61 ± 115
aph(3")-I	116 ± 87	21 ± 28	33 ± 51	60 ± 76
aph(3')-I	113 ± 110	21 ± 34	17 ± 24	55 ± 85
Other	1944 ± 616	447 ± 496	409 ± 338	2294 ± 2003

Table S3. The average relative abundance of ARG subtypes in Shandong, Jiangsu, Zhejiang, Fujian and Guangdong, respectively

Subtypes of ARGs	Shandong (ppm)	Jiangsu (ppm)	Zhejiang (ppm)	Fujian (ppm)	Guangdong (ppm)	Total (ppm)
floR	229 ± 148	168 ± 125	186 ± 423	130 ± 81	119 ± 171	159 ± 238
chloramphenicol exporter	159 ± 72	106 ± 71	130 ± 242	157 ± 146	95 ± 127	126 ± 156
*sul*1	206 ± 148	123 ± 89	108 ± 202	101 ± 62	101 ± 127	118 ± 138
*tet*M	244 ± 117	66 ± 61	99 ± 128	148 ± 170	84 ± 101	115 ± 133
*sul*2	219 ± 128	138 ± 116	55 ± 68	69 ± 44	82 ± 101	99 ± 104
*tet*L	48 ± 24	48 ± 65	80 ± 121	146 ± 199	86 ± 145	86 ± 139
multidrug transporter	51 ± 35	113 ± 100	41 ± 34	79 ± 62	89 ± 106	76 ± 81
*tet*A	73 ± 65	90 ± 107	23 ± 32	67 ± 60	112 ± 159	72 ± 104
aph(6)-I	203 ± 130	87 ± 71	39 ± 53	40 ± 31	57 ± 93	71 ± 90
*aad*A	140 ± 72	84 ± 67	31 ± 38	59 ± 33	69 ± 81	69 ± 67
*acr*B	37 ± 37	108 ± 144	25 ± 34	58 ± 66	89 ± 135	66 ± 104
*tet*W	25 ± 20	33 ± 58	65 ± 111	113 ± 175	60 ± 101	64 ± 117
*erm*B	99 ± 104	20 ± 20	67 ± 131	108 ± 169	30 ± 43	61 ± 115
aph(3")-I	162 ± 102	70 ± 57	35 ± 48	35 ± 27	54 ± 90	60 ± 76
aph(3')-I	170 ± 111	78 ± 94	23 ± 35	20 ± 14	47 ± 86	55 ± 85
Other	1508 ± 370	1129 ± 914	589 ± 566	1138 ± 956	940 ± 1049	2294 ± 2003

Table S4. The top 6 most abundant ARGs of each sample in feces, soil and water, respectively

Origin	Multidrug (ppm)	Tetracycline (ppm)	Aminoglycoside (ppm)	Chloramphenicol (ppm)	MLS (ppm)	Sulfonamide (ppm)
Feces	1969 ± 1398	1147 ± 406	934 ± 471	462 ± 299	535 ± 238	386 ± 261
Soil	165 ± 113	271 ± 313	231 ± 359	392 ± 564	127 ± 223	123 ± 128
Water	285 ± 292	258 ± 264	188 ± 218	103 ± 138	85 ± 135	111 ± 123

Table S5. The top 6 most abundant ARGs of each sample in Shandong, Jiangsu, Zhejiang, Fujian and Guangdong, respectively

Location	Multidrug (ppm)	Tetracycline (ppm)	Aminoglycoside (ppm)	Chloramphenicol (ppm)	MLS (ppm)	Sulfonamide (ppm)
Shandong	534 ± 525	869 ± 233	1360 ± 380	468 ± 215	469 ± 210	426 ± 267
Jiangsu	1364 ± 1572	500 ± 307	475 ± 421	321 ± 214	210 ± 169	262 ± 200
Zhejiang	365 ± 389	429 ± 504	269 ± 337	347 ± 690	243 ± 319	164 ± 261
Fujian	781 ± 737	804 ± 728	452 ± 451	346 ± 225	344 ± 388	172 ± 99
Guangdong	1207 ± 1652	537 ± 545	346 ± 440	269 ± 355	186 ± 215	185 ± 230

Table S6. The abundance of different phyla in ARG-carrying contigs among the feces, soil and water, respectively

Phyla	Feces	Soil	Water	Total
Proteobacteria	522/1119, 46.6%	227/451, 50.3%	439/635, 69.1%	1188/2205, 53.9%
Firmicutes	506/1119, 45.2%	131/451, 29.0%	119/635, 18.7%	756/2205, 34.3%
Bacteroidetes	60/1119, 5.4%	42/451, 9.3%	34/635, 5.4%	136/2205, 6.2%
Actinobacteria	30/1119, 2.7%	49/451, 10.9%	39/635, 6.1%	118/2205, 5.4%
Other	1/1119, 0.1%	2/451, 0.4%	4/635, 0.6%	7/2205, 0.3%

Table S7. Taxonomic assignments of the 847 ARG-carrying contigs at the family level

Family	Contig numbers	Rates
Moraxellaceae	288	34.0%
Enterococcaceae	174	20.5%
Enterobacteriaceae	63	7.4%
Comamonadaceae	49	5.8%
Pseudomonadaceae	34	4.0%
Aeromonadaceae	28	3.3%
Staphylococcaceae	26	3.1%
Leuconostocaceae	18	2.1%
Streptococcaceae	15	1.8%
Clostridiaceae	14	1.7%
Lactobacillaceae	14	1.7%
Planococcaceae	12	1.4%
Erysipelotrichaceae	11	1.3%
Weeksellaceae	11	1.3%
Carnobacteriaceae	9	1.1%
Burkholderiaceae	8	0.9%
Corynebacteriaceae	8	0.9%
Aerococcaceae	7	0.8%
Bacteroidaceae	6	0.7%
Flavobacteriaceae	6	0.7%
Lachnospiraceae	5	0.6%
Other	41	4.8%

Table S8. Taxonomic assignments of the 212 ARG-carrying contigs at the species level

Species	Contig numbers	Rate
Enterococcus cecorum	74	34.9%
Enterococcus faecium	14	6.6%
Erysipelotrichaceae bacterium MTC7	11	5.2%
Acinetobacter baumannii	9	4.2%
Acinetobacter calcoaceticus	9	4.2%
Enterococcus columbae	6	2.8%
*Anaerobium acetethylicum**	5	2.4%
Escherichia fergusonii	5	2.4%
Staphylococcus aureus	5	2.4%
Enterococcus diestrammenae	4	1. 9%
Lactobacillus agilis	4	1.9%
Weissella paramesenteroides	4	1.9%
Bordetella trematum	3	1.4%
Enterococcus aquimarinus	3	1.4%
Proteus mirabilis	3	1.4%
Other	53	25.0%

Table S9. The abundence of human Pathogenic bacteria in feces, soil and water, respectively

Pathogenic bacteria	Feces	Soil	Water	Total
Enterococcus faecium	13	1	0	14
Acinetobacter baumannii	5	2	2	9
Staphylococcus aureus	3	2	0	5
Proteus mirabilis	2	1	0	3
Aerococcus viridans	0	1	1	2
Corynebacterium diphtheriae	0	2	0	2
Escherichia coli	1	1	0	2
Pseudomonas aeruginosa	0	2	0	2
Arcobacter cryaerophilus	0	0	1	1
Streptococcus suis	0	0	1	1
Total	24	12	5	41
